# Graphitic Carbon Nitride as a Nanostimulant in Basil Microgreens: Insights into Growth, Flavonoid Content, and Water Stress Response

**DOI:** 10.1021/acsomega.5c10297

**Published:** 2026-01-28

**Authors:** Gesiely Rosany Costa Resende, Jhon Lennon Bezerra da Silva, Washington Nunes da Silva, Wilian Henrique Diniz Buso, Vania Sardinha dos Santos Diniz, Alex Fabiano Cortez Campos, Atailson Oliveira da Silva, Marcelo Henrique Sousa, Eliane Vieira Rosa

**Affiliations:** † 509282 Federal Institute of Education, Science and Technology Goiano—Campus Ceres, Ceres 76300-000, Goiás, Brazil; ‡ Postgraduate Program in Irrigation in the Cerrado, Federal Institute of Education, Science and Technology Goiano—Campus Ceres, Ceres 76300-000, Goiás, Brazil; § Federal Institute of Education, Science and Technology Goiano—Campus Iporá, Iporá 76200-000, Goiás, Brazil; ∥ Laboratory for Environmental and Applied Nanoscience, UnB—Planaltina Faculty University of Brasilia, Brasilia 73345-010, Distrito Federal, Brazil; ⊥ International Center of Physics, Physics Institute, 28127 University of Brasilia, Brasilia 70910-900, Distrito Federal, Brazil; # Green Nanotechnology Group, University of Brasilia, Brasilia 70910-900, Distrito Federal, Brazil

## Abstract

Graphitic carbon nitride (g-C_3_N_4_) exhibits strong adsorption and photocatalytic activity through reactive oxygen species generation, which also mediate plant stress responses. This study evaluated the effects of g-C_3_N_4_ on growth and secondary metabolites in basil (*Ocimum basilicum* L.) microgreens under water deficit. The nanomaterial was synthesized by calcination and characterized by X-ray diffraction (XRD), Fourier-transform infrared (FTIR), transmission electron microscopy (TEM), scanning electron microscopy-energy dispersive spectroscopy (SEM-EDS), and dynamic light scattering-electrophoretic light scattering (DLS-ELS). Plants were grown under two hydration regimes (50 and 100% water replacement) and five g-C_3_N_4_ concentrations (0–250 mg L^–1^). Foliar applications were performed at 7 and 14 days after sowing, and plants were harvested at day 21. Under 50% water replacement, g-C_3_N_4_ enhanced growth, increasing leaf area by 34% at 100 mg L^–1^, stem length by 23 and 19% at 50 and 100 mg L^–1^, respectively, and root length by 17% at 250 mg L^–1^ compared to the untreated control. Terpenoid profiles remained unchanged, while flavonoid content increased by 49.98% and 42.87% at 100 and 200 mg L^–1^, respectively, under water stress. Overall, g-C_3_N_4_ acted as a modulator of physiological responses, enhancing stress tolerance in basil microgreens.

## Graphical abstract

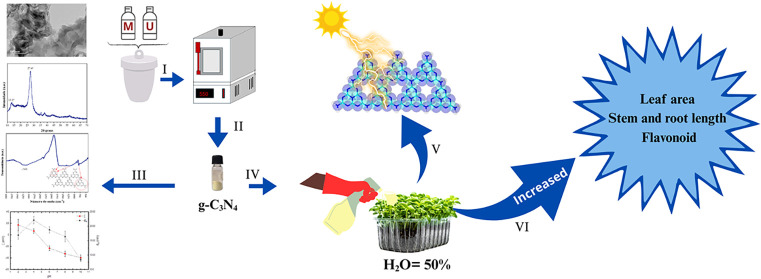

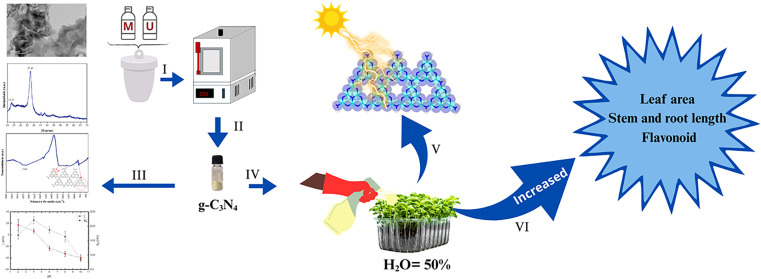

## 1 Introduction

Nanomaterials have gained significant attention in plant science research due to their potential to promote plant growth and enhance the production of nutrients and secondary metabolites.^1–3^ Among them, graphitic carbon nitride (g-C_3_N_4_) emerges as a promising candidate for agricultural applications due to its capacity to generate reactive oxygen species (ROS) under light irradiation in aqueous environments.^4^

Graphitic carbon nitride can be synthesized from diverse precursors and has demonstrated photocatalytic efficiency in degrading organic pollutants, as well as antimicrobial activity against specific microorganisms.^5^ Despite its growing application in environmental remediation, the agricultural potential of g-C_3_N_4_ has been investigated in rice and soybean, but its role in microgreens remains underexplored.^6–8^ Its ability to produce ROS, however, opens new avenues for research in plant stress physiology.

Although ROS were traditionally viewed as merely detrimental byproducts of metabolization, responsible for oxidative damage to biomolecules and cellular structures,^9,10^ emerging evidence has redefined their role in plants. Latest research reveals that ROS function as crucial signaling molecules involved in regulating plant responses to various stressors. By mediating these signaling pathways, ROS help activate defense mechanisms and promote adaptive responses, thereby facilitating plant growth recovery under unfavorable environmental conditions.^11^

Recent whole-season and mechanistic studies show that low-dose g-C_3_N_4_ can act as a nanostimulant. Soil amendments of pristine or Fe-doped g-C_3_N_4_ increased photosynthetic enzyme activities and vegetative cover in rice without yield penalties,^12^ mitigated cadmium- and arsenate-induced phytotoxicity by modulating endophytic communities,^13^ and reduced shoot cadmium migration in soybeans while restoring chlorophyll fluorescence.^14^ Foliar sprays of Fe_2_O_3_/g-C_3_N_4_ enhanced chlorophylls, antioxidant capacity, and fatty-acid profiles in drought-stressed olive trees, effectively raising water-use efficiency under 50% to 75% irrigation regimes.^15,16^ Collectively, these findings position g-C_3_N_4_ as a promising nanoelicitor for both nutrient acquisition and resilience to abiotic stress.

In this context, the present study explores the agricultural potential of g-C_3_N_4_ as a light-responsive nanostimulant by evaluating its effects at varying concentrations on the growth and development of basil (*Ocimum basilicum* L.) microgreens. The investigation focuses on whether subtoxic doses of the nanomaterial can (i) promote seedling growth and tissue hydration, (ii) enhance flavonoid accumulation, and (iii) mitigate the physiological impacts associated with reduced water availability (50% replacement). Basil microgreens were chosen as a model system due to their growing global relevance as a nutrient-dense crop, as they are particularly rich in secondary metabolites, such as polyphenols.^17^ The g-C_3_N_4_ was synthesized via thermal calcination and thoroughly characterized using X-ray diffraction (XRD), Fourier-transform infrared (FTIR), transmission electron microscopy (TEM), scanning electron microscopy-energy dispersive spectroscopy (SEM-EDS), dynamic light scattering (DLS), and electrophoretic light scattering (ELS) techniques. The results point to a potential role of g-C_3_N_4_ in influencing physiological responses in basil microgreens, especially under reduced water availability, suggesting its contribution to plant stress mitigation mechanisms.

## 2 Material and Methods

### 2.1 Synthesis, Processing, and Characterization Methods

X-ray diffraction and FTIR analyses were performed at the University of Brasilia (Nanogreen Laboratory–Faculty of Ceilandia). The X-ray powder diffraction patterns of the g-C_3_N_4_ were recorded on a Miniflex 600 (Rigaku) diffractometer in the range of 2θ = 10° to 90 °Cu–Kα radiation (λ = 1.541 Å). The Fourier Transform Infrared (FTIR) spectra were recorded by a Shimadzu FTIR Spectrophotometer (IRPrestige-21) using conventional KBr:g-C_3_N_4_ pellets in the 4000 cm^–1^ to 400 cm^–1^ range. The morphology of the g-C_3_N_4_ sample was characterized using Transmission Electron Microscopy (TEM) with a JEM-2100 (JEOL) apparatus and Scanning Electron Microscopy (JSM-6610–JEOL) with Energy Dispersive Spectroscopy (SEM-EDS). TEM and SEM-EDS analyses were performed at the Federal University of Goiás (LABMIC–Multiuser High-Resolution Microscopy Laboratory).

The hydrodynamic size (*d*_H_) and zeta potential (ζ) of C_3_N_4_ particles as a function of pH were characterized using Dynamic Light Scattering (DLS) and Electrophoretic Light Scattering (ELS) measurements, respectively, performed with a Malvern Zetasizer Nano ZS90. To prepare the samples, 100 mg L^–1^ of C_3_N_4_ powder was dispersed in aqueous media by sonication in an ultrasonic bath for 15 min under various pH conditions (2, 4, 6, 8 and 10). The ionic strength of the medium was maintained at 10^–2^ mol L^–1^ using NaNO_3_ as the background electrolyte. The hydrodynamic size and polydispersity index (PDI) were determined using the Cumulants expansion method applied to the DLS data. The zeta potential values were calculated from the electrophoretic mobility data using the Smoluchowski approximation.^18^ All experiments were conducted in triplicate at 25 °C, employing Malvern-DTS0012 cuvettes for DLS and Malvern-DTS1070 cuvettes for ELS.

### 2.2 Greenhouse Experiments with Microgreens

The study was conducted in a greenhouse located in the Federal Institute of Education, Science and Technology Goiano (IF Goiano–Campus Ceres), municipality of Ceres in the state of Goiás, Brazil (Latitude 15°18′28″ S, Longitude 49°35′52″ W, and altitude 624 m) (Figure 1), between November and December. Microgreen seeds of the basil variety Padma (Isla—Lots 900320, 900319, 900318, and 162539–883) were selected randomly after mixing the lots. All the seeds were cultivated in Isla production trays (16 cm × 9.5 cm × 5 cm), in a protected greenhouse (70% coverage) with its internal temperature at (25 ± 5) °C and its humidity monitoring performed by a thermohygrometer (Exbom FEPRO 60).

**Figure 1:**
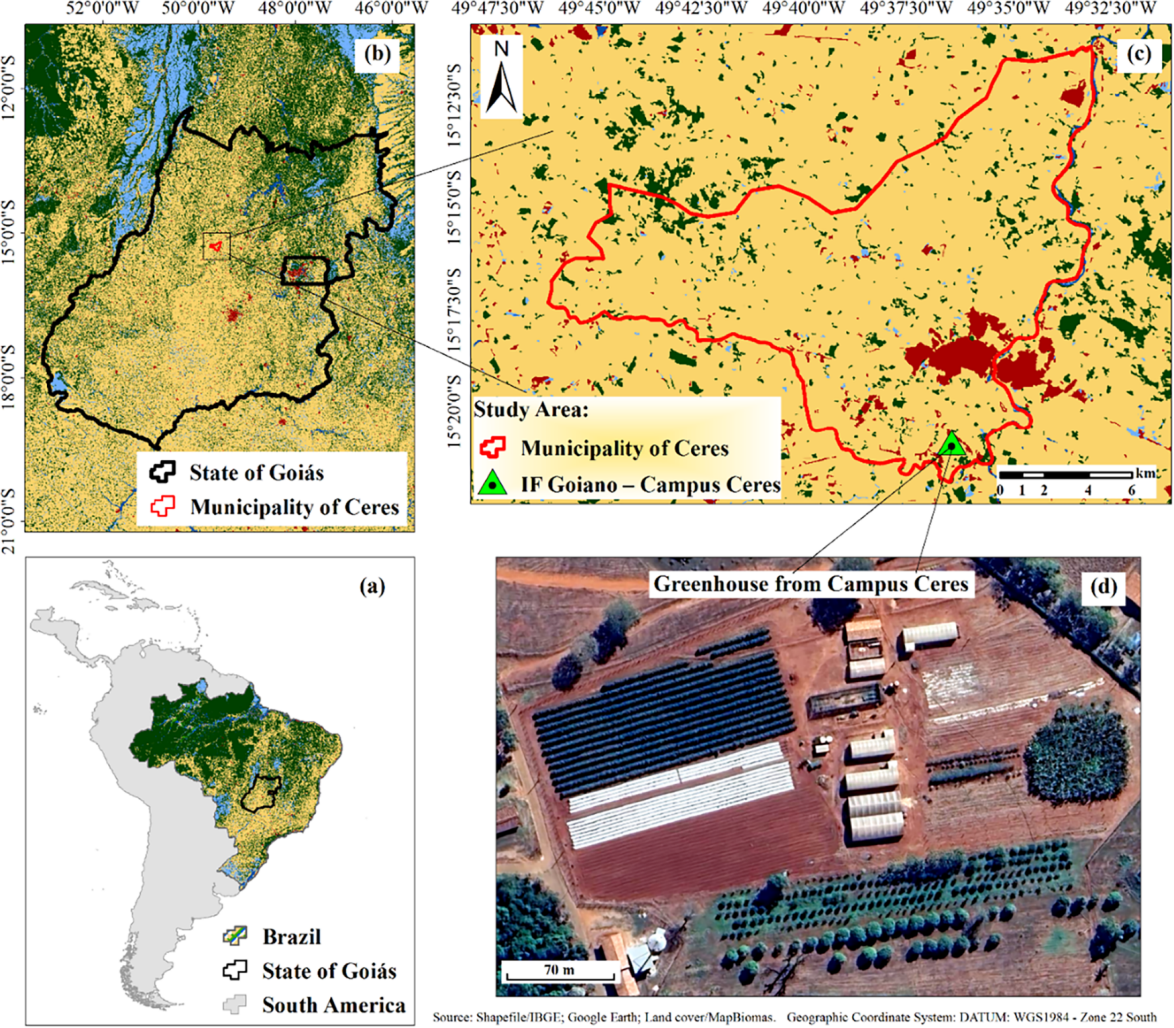
Study area and experimental site: (a–c) maps of Brazil, Goiás State, and IF Goiano–Campus Ceres (MapBiomas Project, Collection 9 of the Annual Land Cover and Land Use Maps of Brazil (1985–2023)), and (d) a view of the greenhouse where the experiments were conducted (Google Earth).

For this experimental stage, the nanomaterial (g-C_3_N_4_) was macerated into a “talc” aspect and dispersed in deionized water in four concentrations mentioned in the methodology and submitted to sonication in an ultrasonic bath (LOGEN) for 30 min. Then, g-C_3_N_4_ was dispersed in deionized water at concentrations of (50, 100, 200, and 250 mg L^–1^). This concentration range was selected based on prior evidence of biological efficacy and growth stimulation in plants exposed to g-C_3_N_4_.^8^

The experimental design applied was a multifactorial 5 × 2: Factor A: carbon nitride dispersed in deionized water (pH 6.0) under five concentrations (0, 50, 100, 200, and 250 mg L^–1^); Factor B: daily water replacement in 2 variations (100% of the recommended by Isla: 25 mL of water; 50% of the recommendation: 12.5 mL of water). For each of the 10 treatments, associating nanomaterial and water replacement, 5 replications were performed, totaling 50 experimental units (each with approximately 200 plants). These units were randomly arranged on benches (spaced 60 cm apart), each containing 5 rows with each experimental unit spaced 12 cm apart.

For 21 days, the growth and commercialization period of microgreen plants recommended by Isla, the experimental units received daily, at 07:00 am, a water replacement of 100% or 50%, according to their treatment, using a manual syringe. The dispersions containing graphitic carbon nitride were applied using an electrostatic sprayer (Generic/Heitign Electrostatic Nanoatomizer: pressure: 280–350 KPA; flow: 22.0 L min^–1^; 35 W) that produces fine mist droplets. Applications were directed to the abaxial (lower) leaf surface to facilitate stomatal penetration, at a spraying distance of approximately 15 cm, and were performed at two stages of the growth cycle: the seventh and 14th days after sowing. The treatments were applied directly to the leaves, sequentially from the lowest to the highest concentration, corresponding to an applied volume of approximately 10 mL per pot.

### 2.3 Analysis of Biometric Parameters

The microgreens samples were harvested manually after 21 days of sowing. The biometric parameters evaluated were leaf area (cm^2^), stem length (cm) and root length (cm). These parameters were obtained through photographs in postharvest and the images were analyzed with the software ImageJ (v1.54d).^19^ The values of the parameters were analyzed for parametric distribution and an ANOVA associated with an *F*-test were subsequently applied, followed by the Scheffé Test for a comparison between the applied treatments, using the OriginLab Pro 2024 Software. The Scheffé test was selected because of its robustness in controlling type I error and its ability to evaluate not only pairwise differences but also linear combinations among treatment groups, even under conditions of reduced statistical power.

### 2.4 Presence of Terpenoids and Analysis of Flavonoids

The samples were subjected to drying in a Forced Circulation Oven (Tecnal) at 50 °C for 3 days.^20^ The dried vegetable samples were macerated and extracted by percolation with 95% ethanol at a 1:5 ratio (g mL^–1^)^21^ for 14 days in amber glass bottles. After this period, extracts from each experimental group were homogenized daily, filtered through sterile gauze, and concentrated using a rotary evaporator (LOGEN) with a 50 °C water bath until complete solvent removal. The resulting residues were then lyophilized.

For the qualitative analysis of terpenoids, thin-layer chromatography (TLC) was carried out using silica gel F254 aluminum plates (Merck). The extract samples were solubilized in 95% ethanol (0.01 g of extract in 1 mL of solvent) and applied to the TLC plates via a glass capillary. The mobile phase consisted of a 8:2 ratio of toluene and ethyl acetate. The plate was visualized under UV light at 365 nm in UV Chamber–Biothec.^22^ The analysis of the content of flavonoids in the ethanolic extracts of each experimental group was performed by Ultraviolet Spectrometry (Bioespectro, model SP220). The standard used was rutin in AlCl_3_ 5% (solubilized in methanol 70%)^23^ at four concentrations: (5, 10, 20, and 30 mg L^–1^), and the absorption measurement was performed at a wavelength of 415 nm.^24^ All samples were prepared at a concentration of 110 mg L^–1^.

## 3 Results and Discussion

### 3.1 Structure and Physicochemical Characterization of g-C_3_N_4_

The synthesized nanomaterial exhibits the appearance of a compact, yellowish powder (Figure 2a), consistent with previous reports on carbon nitride.^7^ The TEM image (Figure 2b) reveals the characteristic morphology of graphitic carbon nitride (g-C_3_N_4_), displaying irregular, layered, plate-like structures formed via thermal polycondensation, in agreement with observations by Rosa and colleagues.^25^ The X-ray diffraction pattern of g-C_3_N_4_ (Figure 2c) exhibits two main peaks at 2θ = 13.1° and 27.4°, corresponding to the (100) and (002) planes, respectively. These peaks are associated with the in-plane structural packing and interlayer stacking of aromatic units, which are typical of g-C_3_N_4_ synthesized from melamine and urea.^26^

**Figure 2:**
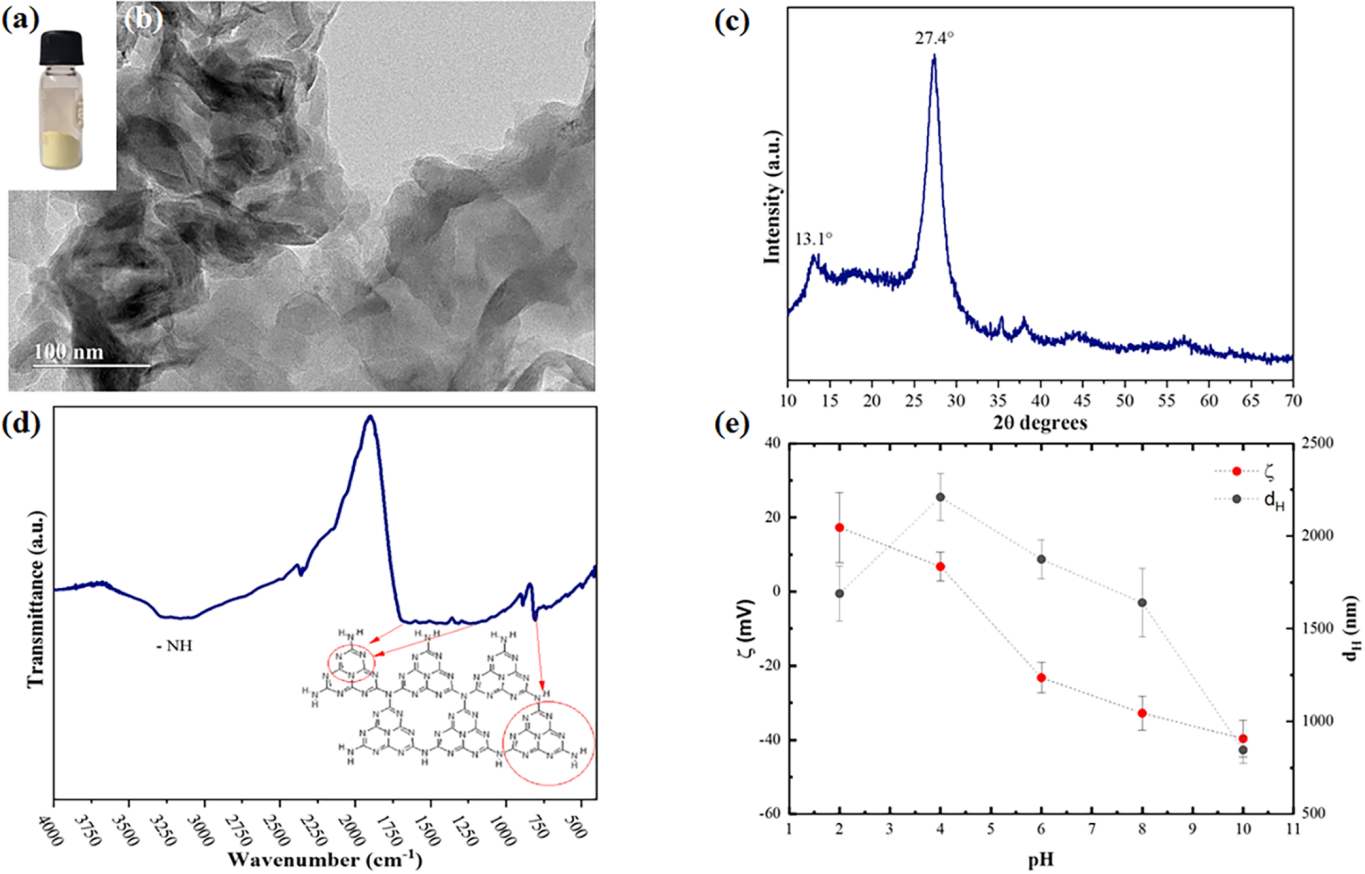
Results of g-C_3_N_4_ characterization: (a) Bulk; (b) TEM Images; (c) XRD results; (d) FTIR; (e) pH dependence of the hydrodynamic size and zeta potential.

The FTIR spectrum (Figure 2d) exhibits the main vibration modes characteristic of the g-C_3_N_4_ phase. The bands in the range of 3400 cm^–1^ to 2850 cm^–1^ are assigned to a residual N–H component corresponding to primary and secondary amines, typical of carbon nitride.^27^ The strong bands from 1700 cm^–1^ to 1200 cm^–1^ correspond to the characteristic vibration of the CN heterocycles,^27^ while the bands located near 810 cm^–1^ are assigned to the out-of-plane vibrations of tri*s* triazine rings.^28^

The zeta potential and hydrodynamic size profiles of the g-C_3_N_4_ particles as a function of pH are presented in Figure 2e. In strongly acidic media, positive ζ values are observed due to the protonation of amino surface sites ( ≡ C–NH_3_^+^). As the pH increases, the concentration of these groups decreases due to deprotonation and hydrolysis, resulting in the formation of ≡C–OH groups. This change causes a gradual reduction in the zeta potential, reaching the isoelectric point (IEP) at approximately pH 4.5, in line with previous studies.^29^ At higher pH levels, the progressive deprotonation of these sites generates ≡C–O^–^ groups, leading to increasing negative ζ values. In terms of hydrodynamic size, the DLS results indicate that the g-C_3_N_4_ phase is dispersed as large clusters across the investigated pH range, as previously reported.^30^ A higher absolute value of the zeta potential, indicating stronger repulsion between clusters, correlates with a smaller hydrodynamic size and, consequently, improved colloidal stability. Indeed, near the isoelectric point (IEP), the g-C_3_N_4_ clusters tend to form large aggregates and gradually sediment. In contrast, at pH levels far from the IEP, colloidal stability is maintained due to sufficiently high zeta potential values.

The scanning electron microscopy (SEM) micrograph of g-C_3_N_4_ reveals a heterogeneous surface topography and irregular morphology, characteristic of materials synthesized at 550 °C, as reported by Chamorro-Posada and colleagues.^31^ The EDS mapping (Figure 3b–f) confirms the presence of carbon (C) and nitrogen (N) elements consistent with the expected composition of g-C_3_N_4_. The elemental maps also indicate a uniform distribution of C and N elements throughout the nanomaterial. Images (Figure 3b–3f) Energy dispersive X-ray spectroscopy (EDS) results with elemental mapping, revealing the typical chemical constituents of g-C_3_N_4_.

**Figure 3:**
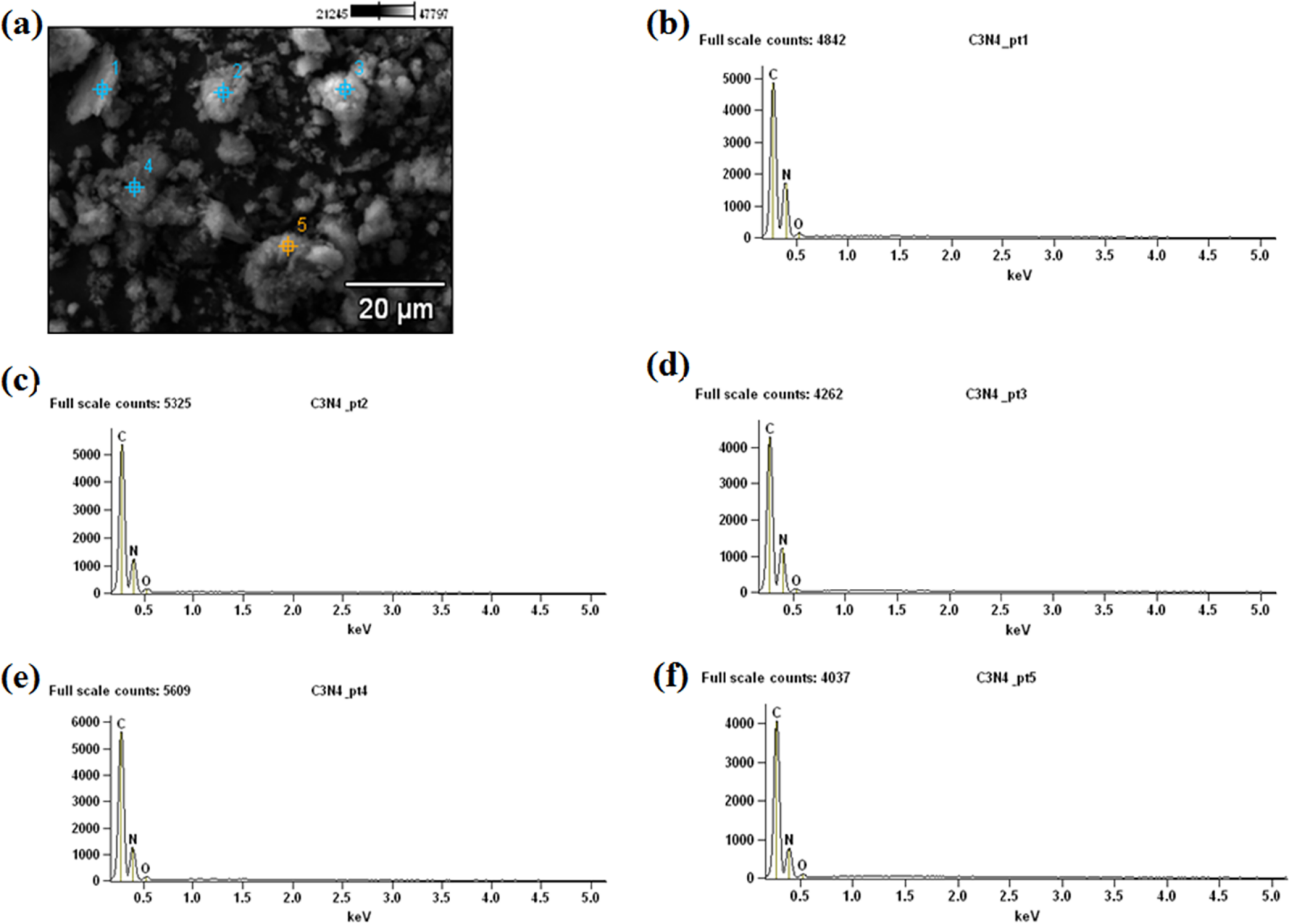
Morphological and elemental characterization of the sample surface: (a) SEM Images; (b–f) energy dispersive X-ray spectroscopy (EDS) results with elemental mapping, revealing the typical chemical constituents of g-C_3_N_4_. Considering the typical precision of SEM–EDS quantification, the compositional values presented here have an estimated uncertainty of roughly 5%.

### 3.2 Biometric Parameters of Microgreens

Analysis of the leaf area parameter under 50% water replacement, using the ANOVA F-test at a 0.05 significance level, revealed significant differences among all treatments. However, subsequent comparisons (Figure 4a) showed no statistically significant difference between the control and the groups treated with 50 mg L^–1^ or 250 mg L^–1^ of the nanomaterial. A significant difference was observed among all other groups, with the highest leaf area recorded in plants treated with 100 mg L^–1^ of the nanomaterial (125.37 mm^2^) compared to the control (82.68 mm^2^). These results suggest that the combination of nanomaterial treatment and water stress enhanced the leaf area of *O. basilicum* L. microgreens, potentially improving their photosynthetic capacity. Conversely, at concentrations above 100 mg L^–1^ and under 50% water replacement, a reduction in leaf area was noted, likely due to a plant response to excessive reactive oxygen species (ROS) generated by the nanomaterial. The phytotoxicity of nanomaterials is known to depend on several factors, including particle size, concentration, and specific plant species. Generally, low concentrations tend to elicit beneficial effects, while higher doses are often associated with toxic responses.^32^

**Figure 4:**
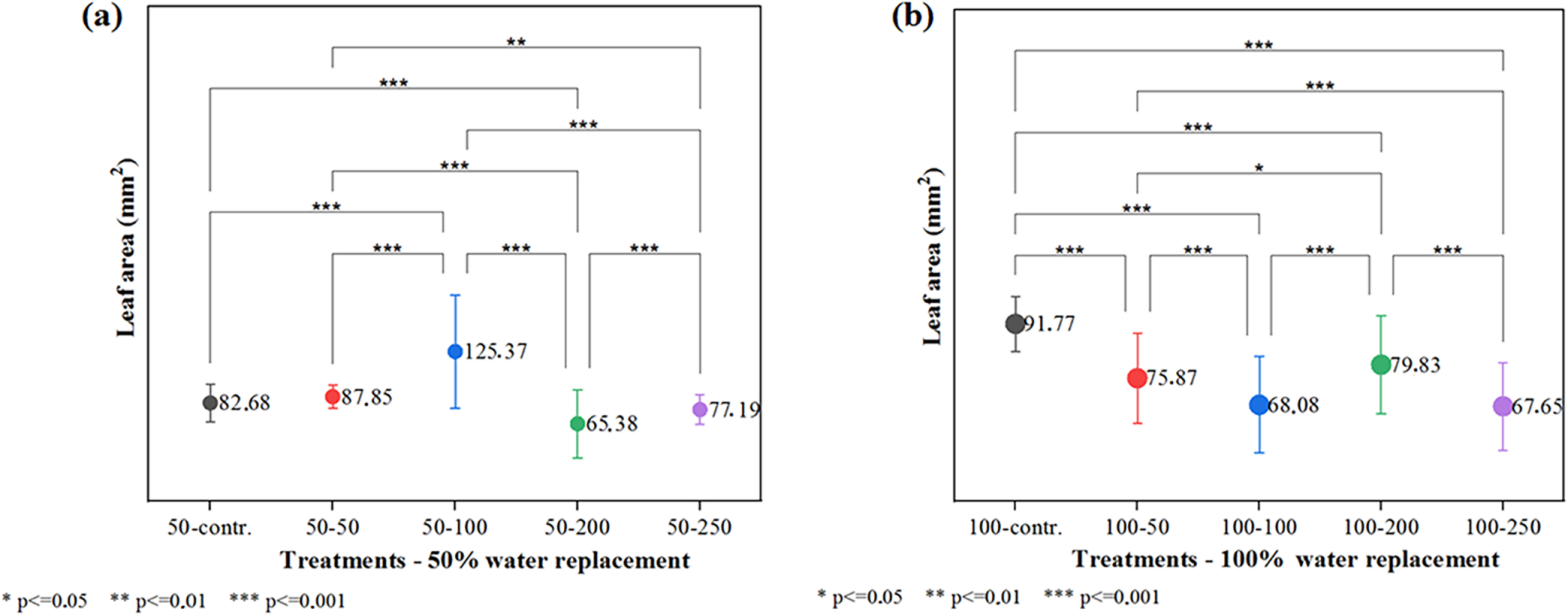
Leaf area analysis: (a) Treatments—50% water replacement combined with 5 nanomaterial treatments; (b) Treatments—100% water replacement combined with 5 nanomaterial treatments.

In the group with 100% water replacement, significant differences in average leaf area were observed among all treatments. However, pairwise comparisons (Figure 4b) indicated that only the groups treated with 100 mg L^–1^ and 250 mg L^–1^ of the nanomaterial did not differ significantly from each other. Compared to the control, all nanomaterial-treated groups exhibited a significant reduction in leaf area. These results suggest that the positive effect of the nanomaterial is more pronounced under water stress conditions. Supporting this observation, Bakry and colleagues reported significantly improved morphological parameters in peanut plants following foliar application of carbon nanotubes at concentrations of 20 mg L^–1^ and 40 mg L^–1^ under water stress levels of 75% and 50%.^33^

In the case of stem length, the experimental group with water replacement indicated significant differences among all treatments in the ANOVA and *F*-test (*p* < 0.05). However, pairwise comparisons (Figure 5a) revealed no significant difference between the control and the group treated with 50 mg L^–1^ of the nanomaterial, nor between the 50 mg L^–1^ group and the others. Notably, treatments with 50 mg L^–1^ and 100 mg L^–1^ resulted in significantly greater stem lengths compared to the control. In contrast, stem length decreased at 200 mg L^–1^ but increased again at 250 mg L^–1^. This nonlinear pattern cannot be fully explained with the current data set. One possible hypothesis is that the reduction at 200 mg L^–1^ may be related to elevated ROS levels and associated oxidative stress, while the subsequent increase at 250 mg L^–1^ could reflect the activation of protective responses and signaling pathways, including phytohormone-mediated processes.^34^ However, these interpretations remain speculative, and further targeted investigations will be required to clarify the mechanisms underlying these contrasting effects.

**Figure 5:**
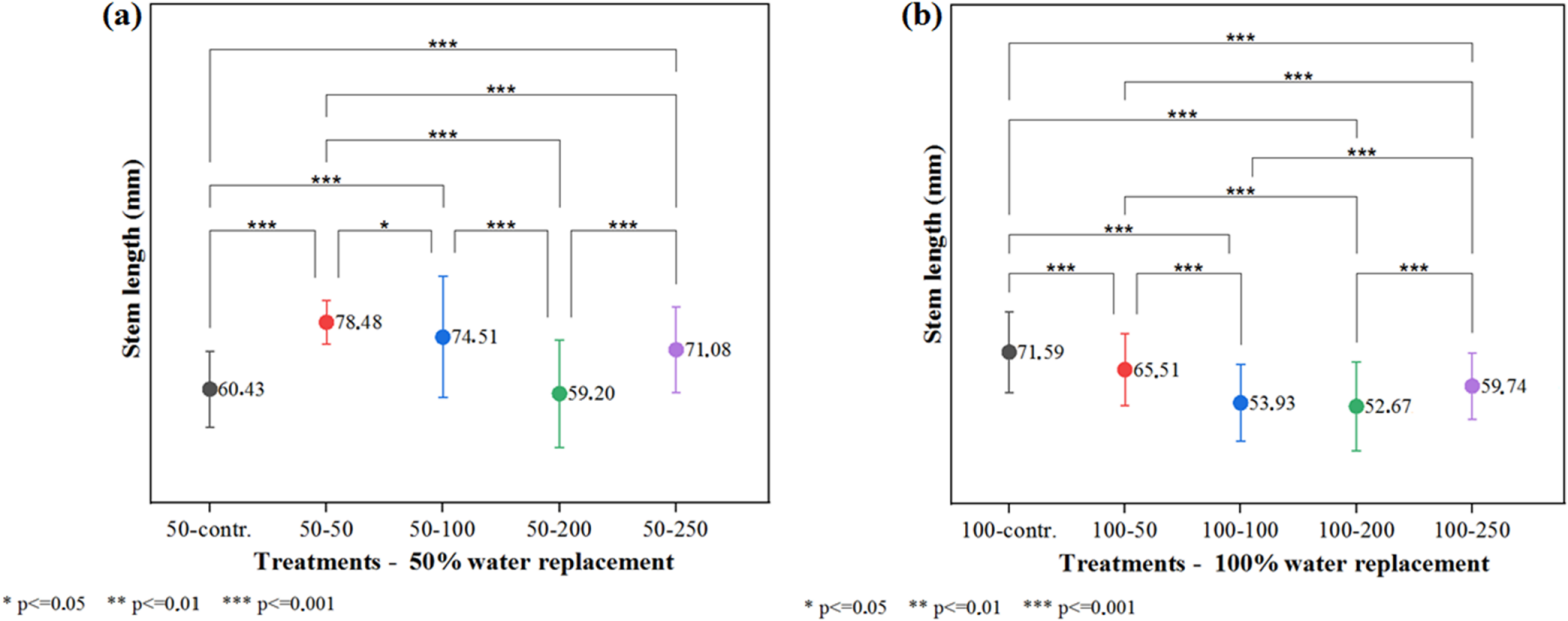
Stem length analysis: (a) Treatments—50% water replacement combined with 5 nanomaterial treatments; (b) Treatments—100% water replacement combined with 5 nanomaterial treatments.

For the experimental groups treated with 100% water replacement, the ANOVA and F-test also confirmed a significant difference among treatments. According to the Scheffé Test (Figure 5b), no significant difference was observed between the groups treated with 100 mg L^–1^ and 200 mg L^–1^. However, all treated groups exhibited significantly lower stem lengths compared to the control. These results are consistent with those observed for the leaf area parameter, reinforcing that the nanomaterial was more effective under conditions of water stress. Similar trends have been reported for other nanomaterials. For example, titanium dioxide (TiO_2_) and sodium nitroprusside were shown to enhance germination and seedling growth in wheat under hydric stress, while showing no beneficial effect under optimal irrigation conditions.^35^ These results support the conclusion that nanomaterials, including the one studied here, may act as growth enhancers when applied under moderate abiotic stress conditions.

For the root length parameter in the 50% water replacement group, the Scheffé Test (Figure 6a) revealed no significant difference between the groups treated with 50 mg L^–1^ and 100 mg L^–1^. However, only the experimental group treated with 250 mg L^–1^ showed a significantly higher mean root length (43.20 mm) compared to the control (36.98 mm). The findings were corroborated by Ma et al.^8^ who, following a 14-day exposure period, demonstrated that a concentration of 250 mg/L of g-C_3_N_4_, synthesized exclusively from urea, significantly promoted rice growth, as evidenced by the superior performance of the treated plants compared to the untreated controls. Under water stress, plants commonly invest in root growth as an adaptive strategy to enhance water and nutrient uptake. Some nanomaterials may function as growth regulators under such conditions, promoting cell wall loosening and expansion, thereby enhancing root development.^35^ In the experimental groups with 100% water replacement (Figure 6b), no significant difference was observed between the groups treated with 100 mg L^–1^ and 250 mg L^–1^. For all other comparisons, statistically significant differences were observed. Nevertheless, all nanomaterial-treated groups exhibited reduced root lengths compared to the control, suggesting that the nanomaterial’s beneficial effects are more pronounced under water-limited conditions.

**Figure 6:**
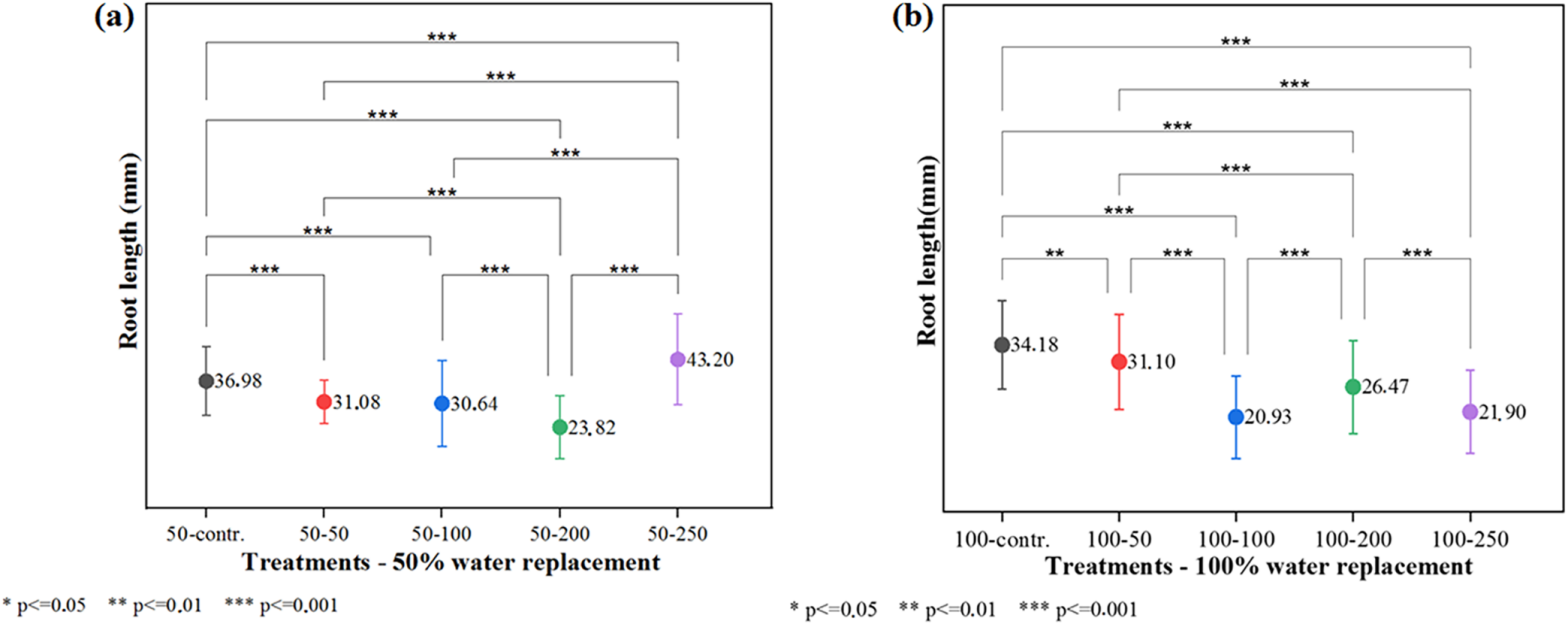
Root length analysis: (a) Treatments—50% water replacement combined with 5 nanomaterial treatments; (b) Treatments—100% water replacement combined with 5 nanomaterial treatments.

The root elongation observed in plants treated with 250 mg L^–1^ under water stress supports the hypothesis that nanomaterials can act as stimulants under abiotic stress. It has been reported that certain nanomaterials can create new pores in plant cell walls, enhancing water and nutrient absorption and promoting root growth.^36^ Additionally, the reactive oxygen species (ROS) generated by some nanomaterials may induce the biosynthesis of stress-related phytohormones such as jasmonic acid, salicylic acid, and abscisic acid, which can elicit positive responses under stress, including enhanced root development.^32^

### 3.3 Presence of Terpenoids and Quantification of Flavonoids

*Ocimum basilicum* L. is well recognized in the food and pharmaceutical sector due to its richness of secondary metabolites, particularly flavonoids and terpenoids, which are key constituents of its essential oils.^37^ The qualitative analysis of terpenoids in the ethanolic extracts of all experimental groups revealed the presence of fluorescent pink bands under UV light (Figure 7). The bands appeared with similar intensities across treatments, indicating a consistent presence of terpenoids under the experimental conditions applied.

**Figure 7:**
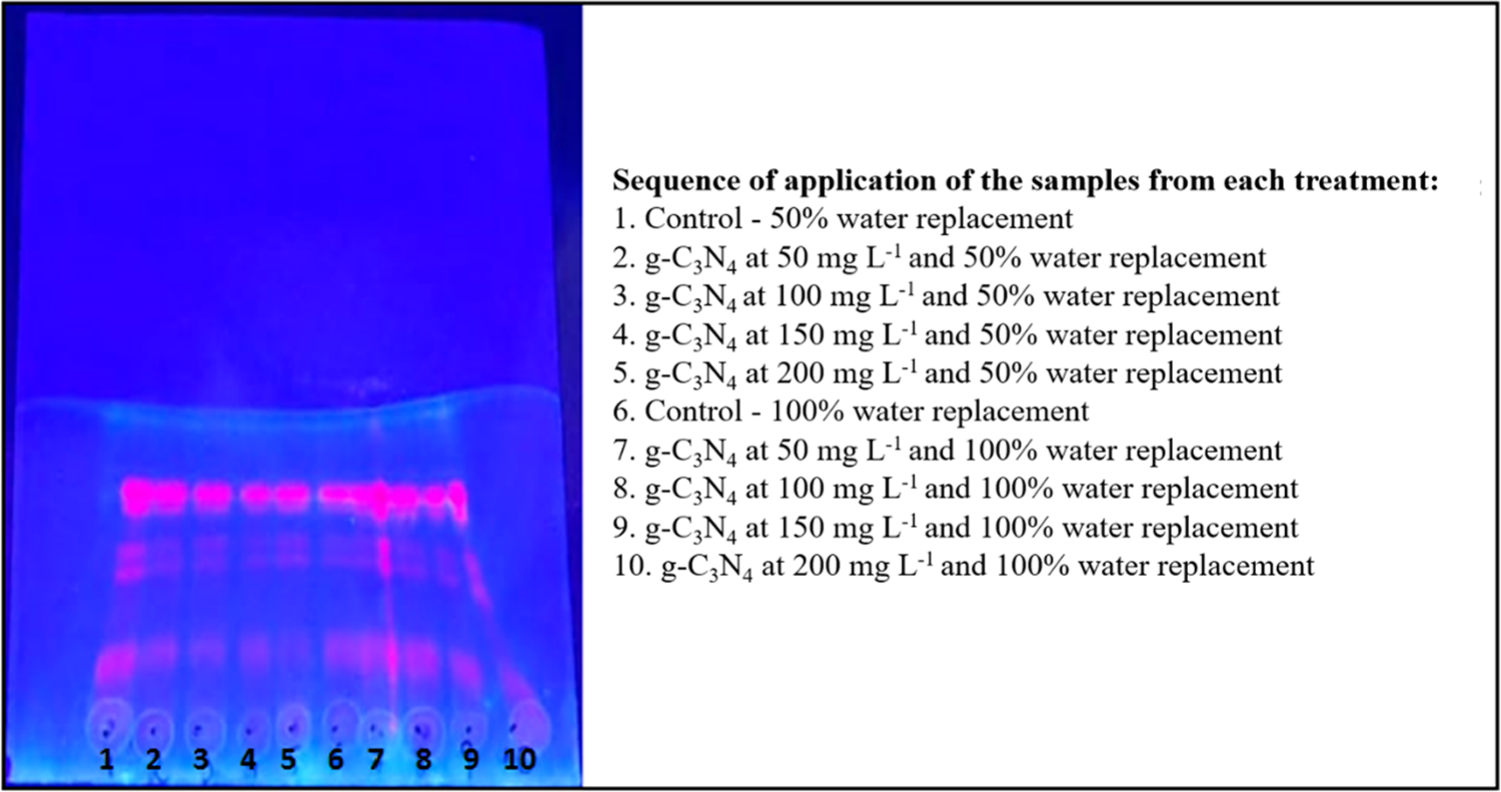
Presence of terpenoids by thin-layer chromatography (TLC), revelation with AlCl_3_ in 70% methanol, exposed to UV light (365 nm). Observe the presence of pink-colored bands, exhibiting uniform intensity and comparable across all treatments.

These results suggest that the nanomaterial, in all tested concentrations, did not qualitatively affect the presence of terpenoids in *O. basilicum* L. extracts, regardless of the water replacement condition (50% or 100%). To better assess the influence of nanomaterials associated with water availability, further quantitative analyses of terpenoid content are recommended.

Regarding total flavonoid content–a group of secondary metabolites highlighted in basil for its antioxidant properties, particularly in response to high ROS levels,^9^ the results (Figure 8b) were similar to those related to the biometric parameters. Under 50% water replacement conditions, an increase in flavonoid content was observed in plants treated with 100 mg L^–1^ and 200 mg L^–1^ of nanomaterial, while a decrease was noted at other concentrations, especially at the highest dose (250 mg L^–1^). In contrast, under 100% water replacement, all nanomaterial-treated groups exhibited lower flavonoid levels compared to the control. This response aligns with the known role of flavonoids as ROS scavengers, particularly under abiotic stress conditions.^38,39^

**Figure 8:**
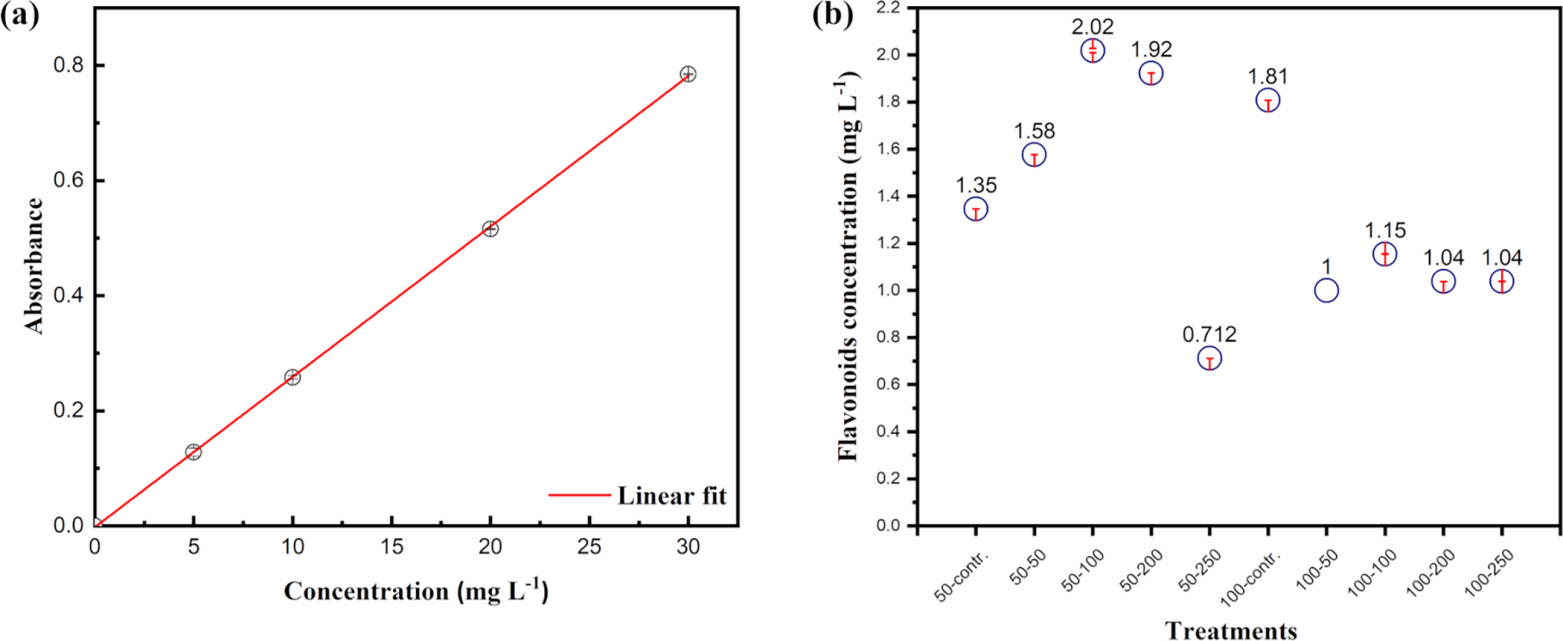
UV Spectrophotometric quantification of flavonoids in *O. basilicum* L.: (a) Calibration curve for standard rutin–aluminum chloride complex spectrophotometrically assayed at 415 nm. The linear regression equation for the calibration curve yielded a correlation coefficient of *R* ^2^ = 0.999. The figures of merit of the instrument were as follows: linear dynamic range of 0.94–30 mg L^–1^, limit of detection of 0.28 mg L^–1^, limit of quantification of 0.94 mg L^–1^, and sensitivity if calibration of 0.026 mg L^–1^; (b) comparative flavonoid concentrations across experimental groups, indicating differential phytochemical accumulation under distinct treatment conditions.

The accumulation of flavonoids in response to stress has been observed in several plant species.^40,41^ One of the responses of flavonoids to stressful environmental factors is the elimination of ROS, as already observed in tea leaflets (*Camellia sinensis*). One of their primary roles is to mitigate oxidative stress by neutralizing ROS, as observed in *Camellia sinensis* (tea leaves). In the present study, the increase in flavonoid content in the 50% water replacement group may have contributed to the improved growth responses (leaf area, stem length, and root development) in nanomaterial-treated plants, possibly by counteracting ROS overproduction.

ROS can play dual roles in plant physiology: at low concentrations, they function as signaling molecules that trigger defense and adaptation mechanisms. However, at sublethal or high levels, they can lead to cellular damage and inhibited growth.^32^ In plants under full irrigation (100% water replacement), the absence of a corresponding increase in flavonoid content may have hindered ROS detoxification, resulting in reduced growth parameters–except for root length, which showed improvement at higher nanomaterial concentrations.

The results suggest that the physiological modulation of *O. basilicum* L. microgreens can be achieved through the foliar application of g-C_3_N_4_ nanomaterials under water stress conditions, as well as their relevance from a nanosafety perspective. Toxicological evaluation using *Danio rerio* (zebrafish) embryos confirmed the absence of acute toxicity for this nanomaterial,^25^ produced by an identical route, thereby reinforcing the biocompatibility of g-C_3_N_4_ in aqueous systems. This offers functional agricultural benefits while minimizing ecological risk.

In this context, considering the results of this nanomaterial in promoting important alterations in agronomic parameters and stimulating flavonoid accumulation under water deficit, without compromising organism viability in aquatic models, carbon nitride can be a promising input for agriculture. Future studies addressing its long-term effects, including physiological mechanisms, interactions with soil and microbiota, and its broader ecotoxicological footprint, will be crucial to validate its deployment in sustainable cropping systems.

Because the experiments were conducted in a controlled, covered greenhouse and involved microgreens with short and uniform growth cycles, environmental variability across experimental rounds was minimized. Although the study did not include additional cultivation cycles in different seasons, the controlled conditions and high degree of environmental standardization support the reproducibility of the observed responses. Nevertheless, we recognize the importance of confirming these findings under broader temporal and environmental scenarios, and future work should include repeated trials across multiple cycles and seasons to further validate the robustness of the agronomic and physiological effects reported here.

## 4 Conclusions

This study presents an initial investigation into the potential use of graphitic carbon nitride (g-C_3_N_4_) as a nanostimulant, with a particular focus on its role in mitigating the effects of water deficit conditions. The results, especially under conditions of 50% water replacement, revealed significant improvements in plant biometric parameters and flavonoid content. These results suggest that g-C_3_N_4_ may function as a metabolic stimulant under moderate water stress, contributing to enhanced plant performance.

In addition, the use of microgreens as a model system in nanomaterial research offers practical advantages, including a short cultivation cycle and increasing global relevance in both production and consumption. This highlights their suitability for rapid screening of nanomaterial effects in plant science.

In conclusion, the data obtained in this study supports the potential of g-C_3_N_4_ as a promising nanomaterial for sustainable agriculture under water-limited conditions. Conducted under controlled greenhouse conditions with minimal environmental variability, the results offer strong indications of reproducibility, although validation across additional growth cycles and seasons remains an important next step. These findings encourage further research across a broader range of plant species and experimental conditions, including detailed physiological and molecular assessments. Such studies could significantly advance the development of nanotechnology-based strategies for resilient and sustainable crop production.
